# Assessing Receptor Activation in 2D and 3D Cultured Hepatocytes: Responses to a Single Compound and a Complex Mixture

**DOI:** 10.3390/toxics12090631

**Published:** 2024-08-28

**Authors:** Laiba Jamshed, Shanza Jamshed, Richard A. Frank, L. Mark Hewitt, Philippe J. Thomas, Alison C. Holloway

**Affiliations:** 1 Department of Obstetrics and Gynecology, McMaster University, Hamilton, ON L8S 4L8, Canada; jamshel@mcmaster.ca (L.J.); shanza.jamshed@gmail.com (S.J.); 2 Water Science and Technology Directorate, Environment and Climate Change Canada, Burlington, ON L7S 1A1, Canada; richard.frank@ec.gc.ca (R.A.F.); mark.hewitt@ec.gc.ca (L.M.H.); 3 Environment and Climate Change Canada, National Wildlife Research Centre, Ottawa, ON K1S 5B6, Canada; philippe.thomas@ec.gc.ca

**Keywords:** cell culture, two-dimensional (2D), monolayer, three-dimensional (3D), spheroids, dexamethasone, naphthenic acids, receptor activation, apoptosis

## Abstract

Responding to global standards and legislative updates in Canada, including Bill S-5 (2023), toxicity testing is shifting towards more ethical, *in vitro* methods. Traditional two-dimensional (2D) monolayer cell cultures, limited in replicating the complex *in vivo* environment, have prompted the development of more relevant three-dimensional (3D) spheroidal hepatocyte cultures. This study introduces the first 3D spheroid model for McA-RH7777 cells, assessing xenobiotic receptor activation, cellular signaling, and toxicity against dexamethasone and naphthenic acid (NA)-fraction components; NAFCs. Our findings reveal that 3D McA-RH7777 spheroids demonstrate enhanced sensitivity and more uniform dose–response patterns in gene expression related to xenobiotic metabolism (AhR and PPAR) for both single compounds and complex mixtures. Specifically, 3D cultures showed significant gene expression changes upon dexamethasone exposure and exhibited varying degrees of sensitivity and resistance to the apoptotic effects induced by NAFCs, in comparison to 2D cultures. The optimization of 3D culture conditions enhances the model’s physiological relevance and enables the identification of genomic signatures under varied exposures. This study highlights the potential of 3D spheroid cultures in providing a more accurate representation of the liver’s microenvironment and advancing our understanding of cellular mechanisms in toxicity testing.

## 1. Introduction

Cell culturing is an important and necessary process in drug discovery, basic science research, and toxicology studies [1,2]. In Canada, recent changes in legislation, including Bill S-5 (2023), have introduced significant changes in risk assessment and management practices to environmental protection, impacting the direction of toxicity testing methods. Globally, toxicity testing is moving from animal-based studies to *in vitro* alternatives, based on the 3R (Replacement, Reduction, and Refinement) approach [3,4,5]. To date, many cytotoxicity and ADME-Tox tests rely on conventional two-dimensional (2D) cell culture [1]. However, 2D cultures are not fully representative of the cellular, molecular, and biochemical geometry of tissues and organs [6]. In 2D cultures, cells are grown on plastic or glass substrates, which alters cell morphology, cellular differentiation and proliferation, cellular polarity [6], extracellular matrix (ECM) protein composition, cellular communication via cytokines and chemokines, and nutrient, oxygen, and pH gradients [7]. In general, three-dimensional (3D) cell culture models can better mimic the *in vivo* microenvironment; they can model cell-to-cell interactions and spatial architecture, and allow for the establishment of nutrient, oxygen, and signaling factor gradients [1,8]. Accumulating evidence in many organ systems suggests that culturing cells in 3D spheroids can re-establish cell–cell contacts and specific microenvironments that allow them to express a tissue-like phenotype by aggregating into large (several hundreds of micrometers) spheroids, providing more physiologically relevant responses for toxicity studies [9].

Multicellular 3D spheroid cultures have been in development since the 1970s [8,10,11] and have been applied to many organ and tissue cell types, including liver hepatocytes. Hepatocytes grown as 2D monolayers have a flat, elongated shape which is distinctly different from their bipolar/stellate shape found *in vivo* [12,13]. Moreover, 2D monolayers of hepatocytes have been shown to have reduced activity and expression of genes involved in drug metabolism [14]. In fact, both activity and gene expression for Phase I and Phase II enzymes (i.e., cytochrome p450s; CYPs) are significantly decreased during the first 24–48 h in monolayer culture [14,15,16]. Additionally, in primary rat hepatocytes cultured as a 2D monolayer, plasma membrane protein distribution [17], canalicular structure, and membrane transport activity [18] are rapidly lost [19]. However, in gel-based systems, as well as spheroid culturing, these ultrastructure morphologies and hepatocellular functions are retained [6,14,19,20].

Within the last decade, 3D culture systems have evolved to extend the cell culture period and retain hepatocyte function. Ranging from monolayer sandwich culture and spheroids to more advanced fluidic, reactor, and perfusion-based systems, 3D culturing techniques have been developed to mimic the *in vivo* microenvironment of the hepatocyte, including its response to endogenous and exogenous substrates [9]. Hepatocytes grown as 3D spheroids have increased interactions with adjacent cells and similar morphology to hepatocytes in tissues [13,20]. They retain the expression of hepatic secretory proteins and phase I and II enzymes [13,21,22] and can develop gradients of oxygen, nutrients, metabolites, and soluble signals, thus creating heterogeneous cell populations (e.g., hypoxic versus normoxic, quiescent versus replicating cells) [8]. This heterogenous mixture of cells allows for well-defined geometry and optimal physiological cell–cell and cell–ECM interactions. As such, 3D spheroids of hepatocytes can create a more realistic morphology and microenvironment for *in vitro* toxicity testing [13,20]. While there is accumulating evidence of function retention in 3D hepatocyte models using various liver constructs and fluidic devices [9,22,23,24,25,26,27], these culture-based platforms have not been assessed for receptor-based toxicology assessments [28,29]. To date, most groups have focused on investigating and validating human cell-based 3D systems [30,31,32,33,34] to investigate drug toxicity (i.e., LD50, IC50) [35]. While there are fewer studies in non-human mammalian models, rat-derived 3D liver models for hepatotoxicity testing have focused on either modelling drug-induced liver injury or their use to support the drug development process [36,37,38,39,40]. There are currently no studies characterizing mammalian 3D liver models and their responses to xenobiotic exposure, specifically environmental pollutants. As such, it is important to recognize the value of rat-derived models in the broader context of generalized mammalian wildlife toxicology. Rat models are crucial for understanding toxicological effects that may impact wildlife health, ecosystem changes, and environmental monitoring. Importantly, characterizing and optimizing culture conditions for 3D spheroids will allow for a better understanding of cellular biology and facilitate the identification of genomic signatures when exposed to environmental stressors either in the form of complex mixtures or individual toxicants.

In this paper, we compare 2D monolayer and 3D spheroid cultures of an immortalized rat hepatocyte model (McA-RH7777 cells) and their responses to the synthetic glucocorticoid dexamethasone, and a highly complex mixture of naphthenic acid fraction components (NAFCs; primarily O_2_ species) extracted [41] from active oil sand process-affected water (OSPW) sampled from a mining operation within the Alberta Oil Sands Region (AOSR). NAFCs are considered the primary toxicant component of OSPW and have been extensively studied in invertebrates and fish; however, there is a significant gap in the understanding of NAFC-induced effects on mammalian wildlife. We investigated xenobiotic receptor activation, cellular signaling and communication, cytotoxicity, and apoptosis in 2D versus 3D spheroid cultures of this cell line.

## 2. Materials and Methods

### 2.1. Cell Culture Maintenance

McA-RH7777 cells, an immortalized rat hepatoma cell line (CRL-1601; ATCC, Manassas, VA, USA) were cultured at 37 °C in a humidified atmosphere of 95% O_2_ and 5% CO_2_ in Dulbecco’s Modified Eagle Media (DMEM 1× with 4.5 g/L glucose, L-glutamine, and sodium pyruvate; DMEM; Corning, Manassas, VA, USA), supplemented with 10% (*v*/*v*) fetal bovine serum (FBS; Gibco, Grand Island, NY, USA), 2 mM L-glutamine, 100 U/mL penicillin, and 100 μg/mL streptomycin (Gibco). Unless otherwise noted, experimental protocols were carried out in DMEM media supplemented as described.

#### 2.1.1. 2D Monolayer Cell Culture

McA-RH7777 cells were grown in 10 mm dishes (Corning). Fresh medium was provided every 48 h, and cells were sub-cultured when ~80% of the cells were fully adhered and cells showed a spindle-like morphology. Cells were considered confluent when 80% of cells were attached to the plate after a minimum of two passages. Confluent cells from passages 12–13 were used for all 2D monolayer experiments.

**Figure 1:**
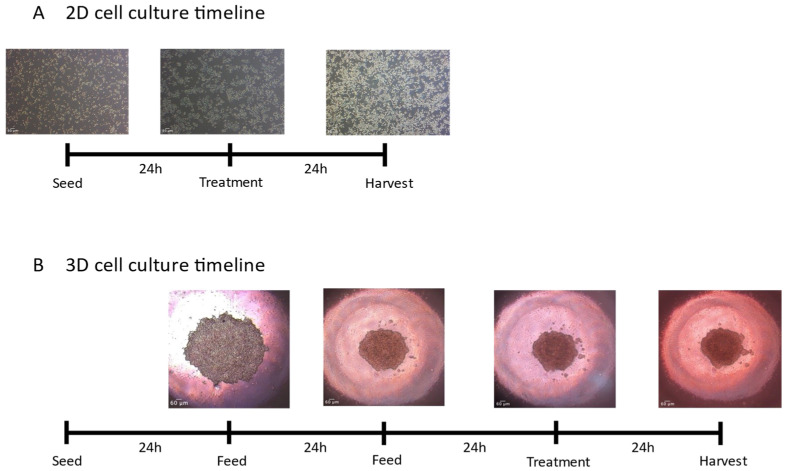
Timeline depicting cell culturing of 2D (**A**) and 3D (**B**) McA-RH7777 cells. Images were taken under a phase-contrast filter and images captured using an AE2000 inverted microscope (Motic, Hong Kong, China) and Moticam X2 camera (Motic). Images of cellular organization were captured at 4× objective magnification. Scales rendered on ImageJ (Fiji, https://imagej.net/software/fiji/ (accessed on 8 December 2023)).

For all assays, a 96-well flat-bottom tissue-culture-treated microplate (Corning) was seeded at a seeding density of 10,000 cells/well (50,000 cells/mL, 250 µL/well). Cells were allowed to adhere for 24 h, after which cells were exposed to treatment-supplemented media for 24 h (Figure 1A). All assays were completed between passages 12–13.

For dose–response exposure experiments, confluent cells from passage 12 were seeded in 6-well plates (Seeding density: 200,000 cell/mL, 2 mL/well; Corning) and grown to 85% confluency prior to 24 h treatment.

#### 2.1.2. 3D Spheroid Cell Culture

McA-RH7777 cells were grown in 10 mm dishes (Corning). Fresh medium was provided every 48 h, and cells were sub-cultured when ~80% of the cells were fully adhered and cells showed a spindle-like morphology. Cells were considered confluent when 80% of cells were attached to the plate after a minimum of two passages. Confluent cells from passages 11–13 were used for all 3D spheroid experiments.

For all assays, an Elplasia^®^ 96-well round bottom ultra-low attachment microplate (Corning) was seeded at a seeding density of 10,000 cells/well (50,000 cells/mL, 200 µL/well). Cells were grown for 3 days (72 h) prior to experimentation (Figure 1B). Supplemented media, as described above, was changed every 24 h by removing 200 µL of media and replacing with 200 µL of fresh media to each well. On day 4, cells were exposed to treatment-supplemented media for 24 h.

For dose–response exposure experiments, an Elplasia^®^ 24-well round bottom ultra-low attachment microplate (Corning) was seeded with 100,000 cells/well in 2 mL of supplemented media, as described above. Cells were grown for 3 days and were fed with supplemented media every 24 h. On day 4, cells were exposed to treatment-supplemented media for 24 h.

We investigated xenobiotic receptor activation, cellular signaling and communication, and cytotoxicity and apoptosis assessments of 2D versus 3D spheroid modelling of this cell line.

### 2.2. Preparation of NAFCs

NAFCs were prepared as previously described in Frank et al. (2006) [41]. Briefly, approximately 2000 L of OSPW was collected from an active tailings pond at Industry A in 2009. The OSPW was stored at 4 °C in the dark until extraction commenced within 6 months of sample collection. NAFCs were extracted, purified, and chemically characterized in Marentette (2015) [42,43]. NAFC extracts were stored in amber bottles at 4 °C.

### 2.3. Cell Viability

McA-RH7777 cells were seeded in 96-well plates (2D: 96-well flat-bottom tissue-culture-treated microplate; 3D: Elplasia^®^ ULA round-bottom 96-well plate; Corning) at a density of 10^5^ cells/mL. Cells were allowed to attach for 24 h and were then treated in duplicate with vehicle (DMEM, control) or NAFCs [0.0025, 0.025, 0.25, 2.5, 1.25, 12.5, 25, 125, 250 mg/L] (*n* = 6 independent experiments) in supplemented DMEM. NAFC treatments were prepared by serial dilution of stock [2504 mg/L] in DMEM supplemented as described above. After 24 h of treatment, cell viability was determined using AlamarBlue^®^ (Bio-Rad Laboratories, Hercules, CA, USA) according to the manufacturer’s instructions. Absorbance was read at 570 and 600 nm using a Synergy H4 Hybrid Reader (BioTek, Winooski, VT, USA).

### 2.4. Cell Culture Treatments

McA-RH7777 cells were treated for 24 h with vehicle (DMEM, control), 1µM dexamethasone, or NAFCs [0.25, 1.25, 12.5, 25, 125 mg/L] (*n* = 6 independent experiments) [41] in supplemented DMEM as described above. Dexamethasone [1 µM] (Sigma, St. Louis, MO, USA), a synthetic glucocorticoid receptor agonist, was selected to assess the effects of GR activation on 2D monolayer and 3D spheroid cultures of McA-RH7777s. There is extensive literature characterizing the effects of glucocorticoids on hepatic function [44,45]. Additionally, it is well established that the glucocorticoid receptor (GR)—when activated—can crosstalk between activated forms of the aryl hydrocarbon receptor (AhR) [46] and hepatic peroxisome proliferator-activated receptors alpha [47] and gamma [48,49] (PPARα and PPARγ). The concentrations of NAFCs [1.25, 25, and 125 mg/L] were chosen to include the range of naphthenic acid concentrations reported in OSPW, surface waters, wetlands, and groundwater from the Alberta Oil Sands Region [42,43,50,51,52,53]. The 24 h exposure represented a timepoint where functional changes in gene exposure were previously observed [54]. Cells were treated as described in Section 2.1.1 and Section 2.1.2.

### 2.5. Real-Time Quantitative PCR

Real-time quantitative polymerase chain reaction (RT-qPCR) was performed to determine changes in gene expression of GR, AhR, PPARα, and PPARγ pathways to examine the impact of 2D versus 3D culture conditions on receptor-mediated changes in xenobiotic metabolism as well as lipid and energy homeostasis, particularly in response to toxicant exposure.

Downstream mRNA expression of genes transcriptionally regulated following activation of AhR [*Cyp1a1*, *Tiparp*], PPARα [*Angptl4*, *Cpt1a*, *Cd36*], PPARγ [*Pgc1α*, *Fabp4*, *Lpl*], and GR [*Sgk1*, *Gilz*] were assessed. In the AhR pathway, *Cyp1a1* (cytochrome p450 family 1 subfamily A member 1) plays a pivotal role in xenobiotic metabolism and serves as a primary responder to environmental toxins [55]. *Tiparp* (TCDD inducible polyADP-ribose polymerase), another AhR target is crucial for modulating the transcriptional activity of AhR and subsequent detoxification processes [56]. We evaluated the downstream target genes of PPARα and PPARγ activation as these pathways are key for nutritional sensing in hepatocytes [48]. Among the genes regulated by PPARα activation, *Angptl4* (angiopoietin-like 4) is a prominent metabolic regulator playing roles in glucose tolerance and lipid homeostasis [57,58,59,60]; *Cpt1a* (carnitine palmitoyl transferase 1A) is a key regulatory enzyme in mitochondrial fatty acid oxidation; and *Cd36* (fatty acid translocase) is a vital transporter for fatty acid uptake [61]. For PPARγ, *Pgc1α* (peroxisome proliferator-activated gamma coactivator 1-α) was included for its role in regulating mitochondrial biogenesis and energy metabolism [62]. Fabp4 (fatty acid binding protein 4), a critical gene in lipid transport and adipocyte function, and Lpl (lipoprotein lipase), which is involved in lipoprotein lipase activity, were also assessed [63]. Lastly, in the GR pathway, Sgk1 (serum glucocorticoid regulated kinase 1) was chosen for its involvement in ion transport and cellular stress responses [64], while Gilz (glucocorticoid-induced leucine zipper) is known for its anti-inflammatory effects and regulation of glucocorticoid responses [65,66,67]. These gene targets were thus integral to comprehensively evaluate the molecular dynamics under the different cell culture conditions and provide insights into the differential regulatory mechanisms of 2D versus 3D cultures.

To assess changes in cellular signaling and communication, we investigated connexins [*Cx26*, *Cx43*]. Connexins [68,69,70] are a class of trans-membrane proteins that create a gap junction between adjacent cells to facilitate intercellular communication and the transfer of ions and small signaling molecules between cells [71]. Additionally, Cx26 is involved in regulation of ATP–calcium signaling, energy supply, invasive capacity, and hepatocellular apoptosis [72], while Cx43 is involved in maintaining tissue homeostasis, controlling cell growth and differentiation, and regulating wound healing [73].

Following cell treatment, total RNA was extracted using TRIzol reagent (Invitrogen, Carlsbad, CA, USA). RNA concentrations were measured using the NanoDrop OneTM Microvolume UV–Vis Spectrophotometer (Thermo Scientific, Waltham, MA, USA). Complementary DNA (cDNA) was synthesized using the High-Capacity cDNA Reverse Transcription Kit (Applied Biosystems, Foster City, CA, USA) as per the manufacturer’s instructions. RT-qPCR was performed using PerfeCta^®^ SYBR^®^ green FastMix^®^ (Quanta Biosciences, Gaithersburg, MD, USA) on the CFX384 TouchTM Real-Time PCR Detection System (Bio-Rad). The cycling conditions included polymerase activation (95 °C for 10 min), followed by 40 cycles of denaturing (95 °C for 15 s) and annealing/elongation (60 °C for 1 min). Levels of gene expression were calculated using the ΔΔCt method [74] and normalized using the geometric means of reference gene expression levels: beta-2-microglobulin (*B2m*) and peptidylprolyl isomerase A (*Ppia*). Primer sequences are supplied in Table 1.

### 2.6. Apoptosis: Caspase 3/7

McA-RH7777 cells were seeded in 96-well plates (2D: 96-well flat-bottom tissue-culture treated microplate; 3D: Elplasia^®^ ULA round-bottom 96-well plate; Corning) at a density of 10^5^ cells/mL to assess apoptosis via caspase 3/7 activity. Cells were allowed to attach for 24 h. After the 24 h attachment period, cells were treated in duplicate with vehicle (DMEM, control) or NAFCs [1.25, 25, and 125 mg/L] (*n* = 8) in supplemented DMEM. Following 24 h of treatment, caspase 3/7 activity was determined using CellEvent^TM^ Caspase 3/7 Green Detection Reagent (Invitrogen, Eugene, OR, USA) according to the manufacturer’s instructions. Fluorescence intensity was measured using a Synergy H4 Hybrid Reader (BioTek).

### 2.7. Statistical Analysis

All statistical analyses were performed using GraphPad Prism (v.9.5.1, GraphPad Software, San Diego, CA, USA). Data were tested for outliers (Grubb’s Test), normality (Shapiro–Wilk), and equal variance. For outcome measures that required comparisons between control and multiple treatment groups, a one-way ANOVA was followed by pairwise comparison using the Dunnett test, where significance was indicated (*p* ≤ 0.05). Data that failed normality were analyzed by Kruskal–Wallis one-way ANOVA on ranks. For comparisons between control and [125 mg/L NAFC] or [1 μM dexamethasone], a Student’s *t*-test was performed. To compare mRNA fold change differences between culture condition (2D confluent and 3D spheroid cultures) and the NAFC dose–response curve, we performed a two-way ANOVA with Sidak’s multiple comparisons test, using a single-pooled variance. All data are presented as mean ± SEM and were considered significant when *p* ≤ 0.05.

## 3. Results

### 3.1. Morphology

McA-RH7777 cells have been used as a model system to determine liver physiology, metabolism, and drug metabolism. As an adherent cell line, under a light microscope, they appear polygonal or cuboidal, and form well-defined monolayers with tightly packed cells (Figure 1A). When grown in ULA wells (Figure 1B), McA-RH7777 cells self-assembled and formed tight spheroids with smooth edges by 24 h (Figure 1B). Spheroids were grown for three days.

### 3.2. D-Cultured McA-RH7777s Exhibited Significantly Higher Fold Change of Altered Genes Following Dexamethasone Exposure

**Figure 2:**
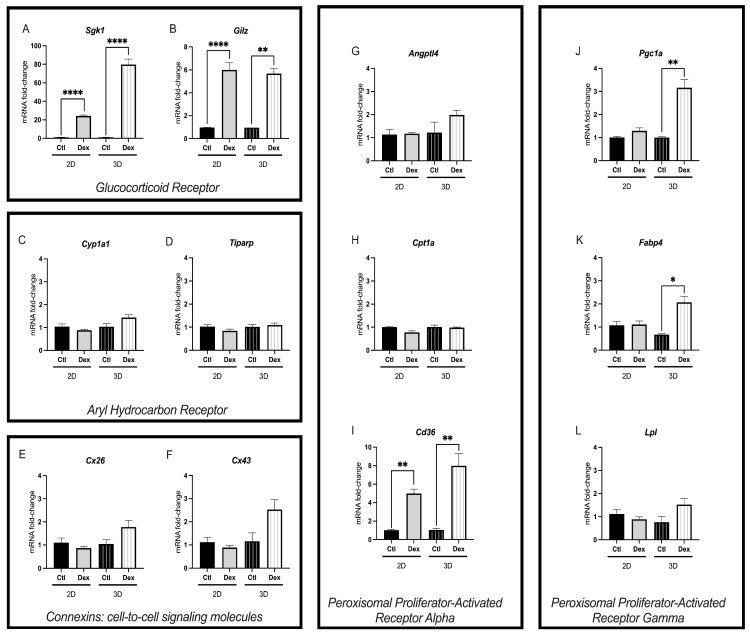
Impact of dexamethasone on mRNA expression of critical pathways involved in xenobiotic metabolism. mRNA expression of genes involved in glucocorticoid signaling: *Sgk1* (**A**) and *Gilz* (**B**); aryl-hydrocarbon receptor activation: *Cyp1a1* (**C**) and *Tiparp* (**D**); connexins, cell-to-cell signaling molecules: *Cx26* (**E**) and *Cx43* (**F**); peroxisomal proliferator activating receptor alpha activation: *Angptl4* (**G**), *Cpt1a* (**H**) and *Cd36* (**I**); and peroxisomal proliferator activating receptor gamma activation: *Pgc1a* (**J**), *Fabp4* (**K**), and *Lpl* (**L**) of McA-RH7777 cells following 24 h exposure to 1μM dexamethasone. Values are shown as mean ± SEM, *n* = 6; asterisks *, **, **** indicate significant differences (*p* < 0.05, 0.01, 0.001, respectively) versus control.

Dexamethasone exposure induced *Sgk1* and *Gilz* mRNA expression in confluent and spheroid cultures (Figure 2A,B). While *Gilz* fold change remained similar between 2D confluent and 3D spheroid cultures (~6 mRNA fold change), *Sgk1* had a significantly higher fold change in 3D culture conditions (two-way ANOVA *p* < 0.0001) compared to 2D confluent cultures. Similarly, while dexamethasone exposure induced the PPARα-transcriptionally regulated gene *Cd36* in both culture conditions (Figure 2I), 3D spheroids had a significantly higher fold change (two-way ANOVA *p* = 0.003). Interestingly, while PPARγ-regulated genes *Pgc1*α, and *Fabp4* were not significantly induced (*p* > 0.05) by dexamethasone exposure in 2D monolayers of McA cells, in 3D cultured spheroids, *Pgc1*α and *Fabp4* mRNA expression was significantly increased (Figure 2J,K). Dexamethasone treatment did not alter the gene expression of any targets related to AhR activation (Figure 2C,D) or connexins (Figure 2E,F).

### 3.3. NAFCs Did Not Significantly Affect Cell-Viability in 2D or 3D Cultures

**Figure 3:**
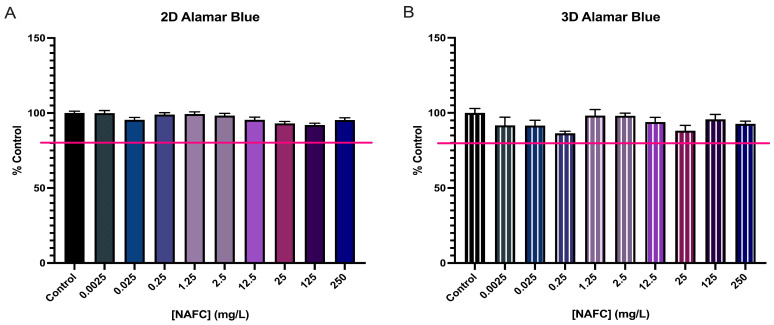
Cytotoxicity assay of 2D (**A**) and 3D (**B**) cultured McA-RH7777 cells following NAFC exposure [0.0025–250 mg/L] (*n* = 6) for 24 h. Viability of 80% is signified by the red line.

Cell viability remained above an 80% threshold (ISO10993-5) in both 2D and 3D cultures in all treatment groups following 24 h exposure to NAFCs (Figure 3).

### 3.4. Exposure to NAFCs Elicits Differential Sensitivity in mRNA Expression of Genes Involved in Receptor Activation between 2D and 3D Cultured McA-RH7777s

**Figure 4:**
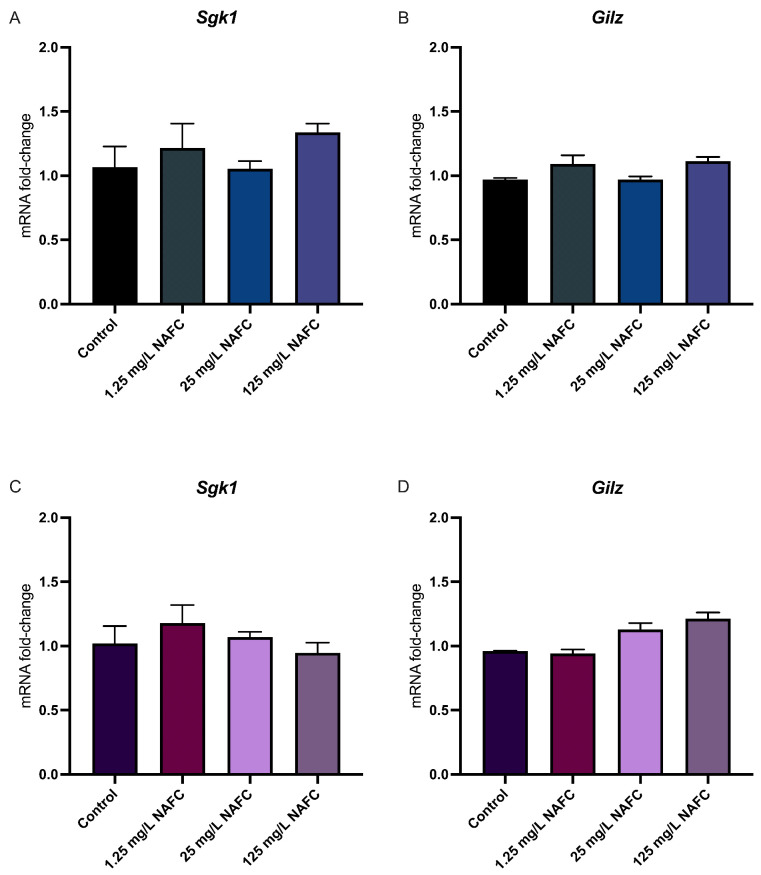
mRNA expression of genes involved in glucocorticoid signaling. *Sgk1* (**A**,**C**) and *Gilz* (**B**,**D**) in 2D (top panel; (**A**,**C**)) and 3D (bottom panel; (**B**,**D**)) cultured McA-RH7777 cells following 24 h exposure to NAFCs [0, 1.25, 25, 125 mg/L] (*n* = 4–6). Values are shown as mean ± SEM.

**Figure 5:**
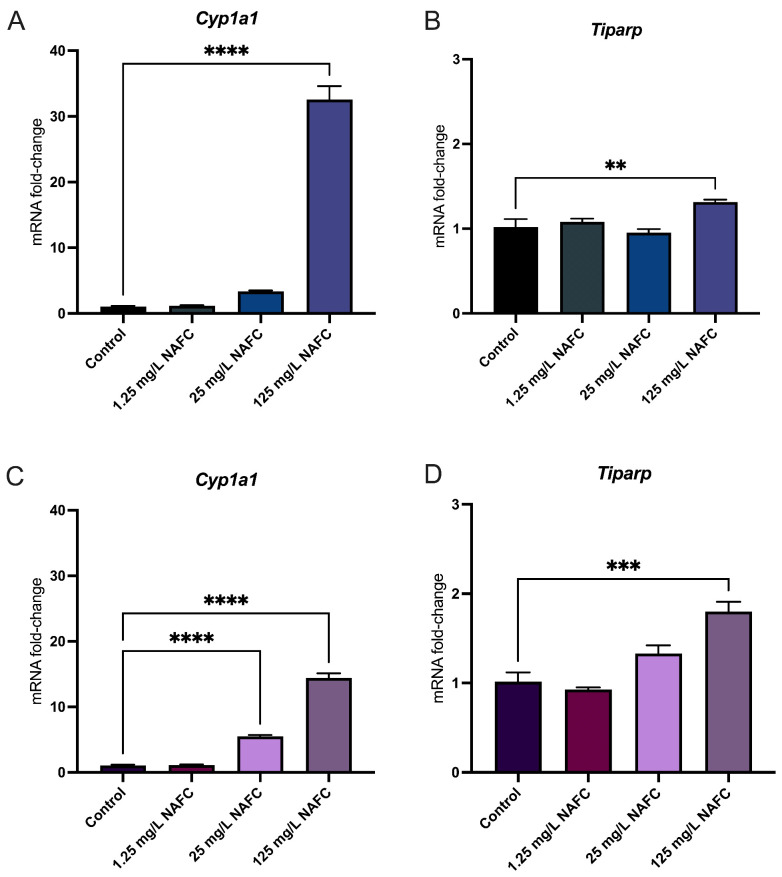
mRNA expression of genes involved in aryl-hydrocarbon receptor activation. *Cyp1a1* (**A**,**C**) and *Tiparp* (**B**,**D**) in 2D (top panel; (**A**,**B**)) and 3D (bottom panel; (**C**,**D**)) cultured McA-RH7777 cells following 24 h exposure to NAFCs [0, 1.25, 25, 125 mg/L] (*n* = 4–6). Values are shown as mean ± SEM; asterisks **, ***, **** indicate significant differences (*p* < 0.01, 0.005, 0.001, respectively) versus control.

**Figure 6:**
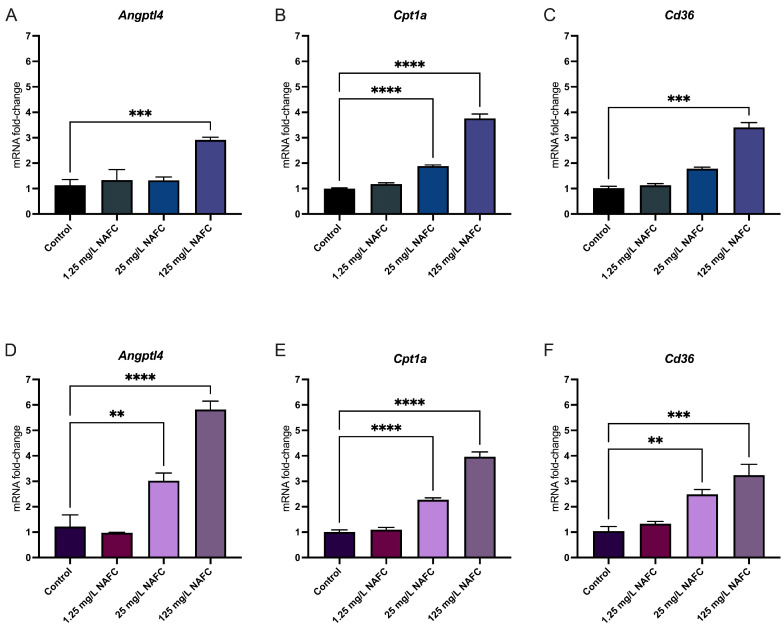
mRNA expression of genes involved in peroxisomal proliferator activating receptor alpha activation. *Angptl4* (**A**,**D**), *Cpt1a* (**B**,**E**), and *CD36* (**C**,**F**) in 2D (top panel; (**A**–**C**)) and 3D (bottom panel; D, E, F) cultured McA-RH7777 cells following 24 h exposure to NAFCs [0, 1.25, 25, 125 mg/L] (*n* = 4–6). Values are shown as mean ± SEM; asterisks **, ***, **** indicate significant differences (*p* < 0.01, 0.005, 0.001, respectively) versus control.

**Figure 7:**
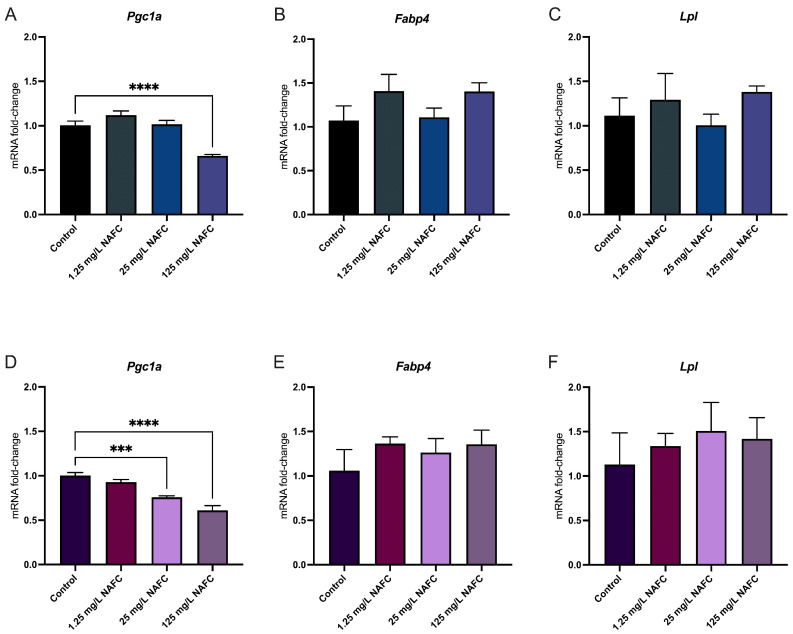
mRNA expression of genes involved in peroxisomal proliferator activating receptor gamma activation. *Pgc1a* (**A**,**D**), *Fabp4* (**B**,**E**), and *Lpl* (**C**,**F**) in 2D (top panel; (**A**–**C**)) and 3D (bottom panel; (**D**–**F**)) cultured McA-RH7777 cells following 24 h exposure to NAFCs [0, 1.25, 25, 125 mg/L] (*n* = 4–6). Values are shown as mean ± SEM; asterisks ***, **** indicate significant differences (*p* < 0.005, 0.001, respectively) versus control.

**Figure 8:**
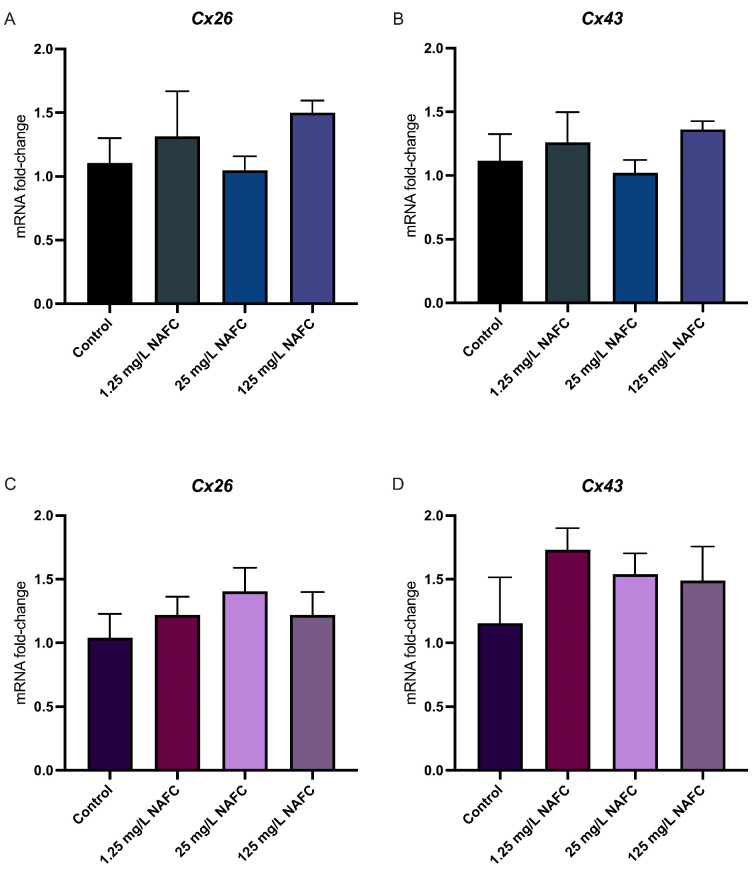
mRNA expression of connexin-genes. *Cx26* (**A**,**C**) and *Cx43* (**B**,**D**) in 2D (top panel) and 3D (bottom panel) cultured McA-RH7777 cells following 24 h exposure to NAFCs [0, 1.25, 25, 125 mg/L] (C, D, G, H) (*n* = 4–6). Values are shown as mean ± SEM, *n* = 6.

**Figure 9:**
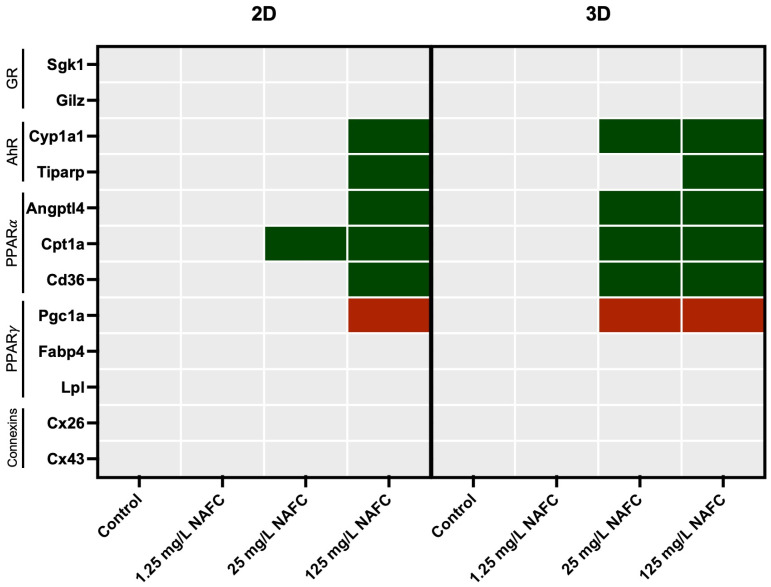
Summary heatmap of receptors: glucocorticoid receptor (GR), aryl hydrocarbon receptor (AhR), peroxisome proliferator-activated receptor alpha (PPARα), peroxisome proliferator-activated receptor gamma (PPARγ)—and connexins. mRNA fold changes in 2D and 3D cultures of McA-RH7777 following 24 h exposure to NAFCs [0, 1.25, 25, 125 mg/L] (*n* = 4–6). Significant inductions are marked in green, while significant decreases are marked in red.

In general, 3D cultured spheroids exhibited a more distinct dose–response relationship across all receptor pathways tested, compared to 2D monolayers (Figure 4, Figure 5, Figure 6 and Figure 7). NAFC exposure did not alter GR-activated gene *Sgk1* or *Gilz* mRNA expression in 2D or 3D cultures (Figure 4). AhR-activated genes *Cyp1a1* and *Tiparp* were significantly induced following exposure to 125 mg/L NAFCs in all cell culture conditions (Figure 5). Interestingly, *Cyp1a1* expression in 2D confluent culture had a significantly higher mRNA fold change compared to 3D spheroids (Figure 5A,C; *p* < 0.0001). Similarly to what we observed with AhR regulated genes, PPARα-transcriptionally activated genes *Angptl4*, *Cpt1a*, and *Cd36* were all significantly induced following exposure to 125 mg/L NAFCs in all cell culture conditions (Figure 6). Additionally, 3D spheroid cultures treated with 25mg/L NAFCs showed a significant increase in mRNA expression of *Cyp1a1*, *Angptl4*, and *Cd36*, an effect which was not observed in 2D confluent culture (Figure 5C and Figure 6D,F). This trend was also observed in the expression of *Pgc1α*, a PPARγ-transcriptionally regulated gene. Indeed, *Pgc1α* mRNA was significantly decreased following exposure to 25 and 125 mg/L NAFCs in 3D spheroid cultures (Figure 7D), whereas we only observed a decrease in its expression following exposure to 125 mg/L NAFCs in 2D confluent cultures (Figure 7A). NAFC exposure did not alter the mRNA expression of any connexins under any culture conditions (Figure 8). The summary heatmap (Figure 9) highlights significant mRNA fold changes of key receptors and connexins in 2D and 3D cultures of McA-RH7777 cells after NAFC exposure.

### 3.5. 3D Cultured McA-RH7777s Are More Resistant to NAFC-Induced Caspase 3/7 Activity

**Figure 10:**
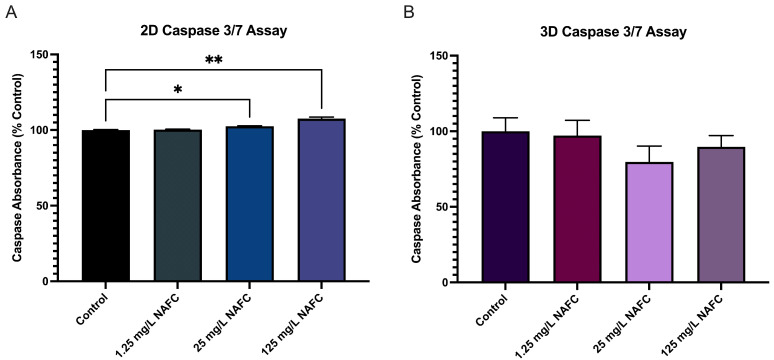
Caspase 3/7 activity in 2D (**A**) and 3D (**B**) McA-RH7777 cells following NAFC exposure [0, 1.25, 25, 125 mg/L] (*n* = 9) for 24 h. Values are shown as mean ± SEM; asterisks *, ** indicate significant differences (*p* < 0.05, 0.01, respectively) versus control.

Although NAFC exposure [25 and 125 mg/L] in 2D cultured McA-RH7777 cells induced caspase 3/7 activity, NAFC exposure in 3D spheroids showed no change (Figure 10).

## 4. Discussion

The goal of this study was to investigate the utility of a 3D rat spheroid culture to assess responses to a single toxicant and a complex mixture. Importantly, while there is a considerable literature available on 3D spheroid models for human health assessment, none have yet been utilized as surrogates for mammalian exposures to environmental toxicants.

Our study is the first to generate a 3D spheroid *in vitro* model for McA-RH7777 cells. These cells, derived from a rat hepatoma, have been extensively used in drug metabolism studies and drug toxicity testing because they mimic many characteristics of hepatocytes and hepatocellular carcinomas. Importantly, our use of a micropatterned plate (Elplasia^®^) for cell-culture dose–response treatments and an ultra-low-attachment (Elplasia^®^) plate for assays offers several advantages including geometric control of cell growth and viability, dynamic imaging of the spheroids, and high-resolution and low-fluorescence assay ability. Here, we have optimized cell culture conditions of McA-RH7777 cells in a 3D environment, allowing for a better understanding of cellular biology and the identification of genomic signatures when exposed to both a complex mixture (NAFC) and a single compound (dexamethasone). Results from this study demonstrate that 3D McA-RH7777 spheroids reveal increased sensitivity and more consistent dose-dependent relationships in gene expression for key components of xenobiotic metabolism responses to exogenous compounds.

### 4.1. Dexamethasone: A Model Corticosteroid

Corticosteroids, the synthetic analogues of endogenous glucocorticoid hormones, induce extensive biochemical changes in the liver, altering many critical processes required for energy homeostasis including gluconeogenesis and lipid metabolism [75,76]. Dexamethasone, a synthetic glucocorticoid, has been extensively studied for its effects on many liver models: *in vitro* cell-lines, *in vitro* primary cultures, and stem cells in organoid culture systems [77,78]. In these models, dexamethasone has been shown to promote hepatocyte differentiation and maturation, modulate cell proliferation, and enhance the expression of liver-specific genes such as albumin, cytochrome P450 enzymes [55], and transporters. While there are no studies on the effect of dexamethasone on 3D cultures of liver cells, dexamethasone has been known to strongly influence the expression of several enzymes involved in metabolism of exogenous chemicals [78].

In our study, dexamethasone treatment of 2D monolayers and 3D spheroids resulted in the induction of GR transcriptionally regulated genes *Sgk1*, and *Gilz*. Sgk1 and Gilz have been associated with various cellular processes in the liver, including adipogenesis, insulin resistance, and hepatic ceramide synthesis—all critical processes in hepatic metabolism [79]. Notably, we observed a higher mRNA fold change for *Sgk1* in 3D cultured spheroids versus 2D monolayers. This observed increased mRNA fold change in 3D cultures versus 2D culture was consistent across other genes which are transcriptionally regulated by PPARγ and PPARα. *Cd36*, a fatty acid translocase with a high affinity for lipid and lipid-containing ligands, was significantly induced in both 2D and 3D cultures, with a significantly higher mRNA fold change in 3D cultures. *Pgc1α* and *Fabp4* were only observed to be significantly induced by dexamethasone exposure in the 3D spheroids. Interestingly, all three of these genes are also regulated by glucocorticoids. For example, *CD36* has functional glucocorticoid receptor binding regions [61] on its sequence, while *Pgc1α* is a well-established co-regulator of GR-mediated gene expression by binding to the ligand binding domain of GR to activate downstream transcriptional factors [62]. *Fabp4* has been found to be upregulated by exogenous glucocorticoid treatment. Additionally, specific GR-nuclear localization and GR-nuclear exposure signal sequences have been identified in the potential functional domains of *Fabp4* [63,80,81]. Notably, there was also a trend of non-statistical significance of increased mRNA fold change in 3D versus 2D culture following dexamethasone exposure (*Lpl p* = 0.0677, *Cyp1a1 p* = 0.0896). Together, these data suggest that 3D culture may have heightened sensitivity or a more robust response to ligand activation of PPARα and AhR.

The increased sensitivity of 3D cultures has been attributed to their ability to provide a more immersive and complex physical structure, resembling the *in vivo* environment, allowing for improved direct cell-to-cell communication due to increased paracrine (i.e., release of signaling molecules between neighboring cells) and juxtracrine (i.e., direct contact-dependent interaction between cell surface receptors and ligands) signaling. In our study, connexin 26, predominantly located in hepatocytes, and connexin 43 [82], expressed in non-parenchymal liver cells, both exhibited a similar trend of increased mRNA fold changes in 3D versus 2D cultures following exposure to dexamethasone; however, these changes did not reach statistical significance (*Cx26 p* = 0.1108; *Cx43 p* = 0.0675). Interestingly, the expression of *Cx43* has been shown to be higher in 3D cultured pancreatic endocrine cells compared to 2D cultured cells [83]. As such, future work should continue to elucidate the impact of culture conditions on connexin expression to enhance our understanding of cellular communication and behavior in varying microenvironments.

As we initially examined the transcriptional regulation of xenobiotic and energy metabolism in hepatocytes under 2D versus 3D culture conditions using a known ligand, we further extended this analysis to include a highly complex environmental mixture of bitumen-derived organics. Environmental exposures that affect wildlife health are typically complex mixtures rather than single compounds.

### 4.2. Naphthenic Acid Fraction Components: An Environmental Mixture

Within bituminous oil sands are a large and diverse group of organic acids that include naphthenic acid fraction components (NAFCs). NAFCs are a complex mixture found in oil sand process-affected water and are of significant environmental concern due to their potential toxicity to aquatic biota; however, few studies have investigated the effects of NAFCs in mammals [84,85]. Our group has previously shown that NAFCs have altered critical cell-stress pathways mediated by receptor activation in murine osteoblasts [86] and human placental trophoblasts [54]. Additionally, there is evidence that compounds found in OSPW and other unconventional oil and gas extraction methods can act as endocrine-disrupting compounds by affecting glucocorticoid production in aquatic species [86,87,88]. Therefore, 2D monolayers and 3D spheroid cultures of hepatocytes were treated with NAFCs to assess the same transcriptional targets of xenobiotic and energy metabolism.

In McA-RH7777 cells exposed to NAFCs, there was no change in the dynamic range of mRNA expression between 2D and 3D cultured cells, as observed with dexamethasone treatment, a known GR-agonist, nor did NAFC exposure alter GR-activated genes in 2D confluent or 3D spheroid cultures. NAFC exposure significantly altered mRNA expression of genes involved in AhR, PPARα, and PPARγ response genes. Notably, while 2D monolayers exhibited altered mRNA expression, primarily in response to the highest concentration of 125 mg/L NAFCs; a lower concentration of 25mg/L NAFCs was also effective in causing significant changes in mRNA expression of genes transcriptionally regulated via AhR (*Cyp1a1*), PPARα (*Angptl4*, *Cd36*), and PPARγ (*Pgc1α*) in 3D culture. Interestingly, many of these genes can be co-regulated by additional receptor activation. For example, *Cyp1a1*, a known marker of AhR-ligand activity, has been found to have two PPAR element sites on its promotor region, which can be activated by PPARα ligands [89]. Similarly, Pgc1α, regulated by PPARγ, has also been found to be modulated by GR and AhR-ligands. Additionally, Angptl4 is known to be regulated by various PPAR isoforms, including PPARα, PPARγ, and PPARβ/δ [90,91], in response to changes in physiological states (i.e., fasting, feeding) [92] and exposure to environmental stressors [93]. Complementing these findings, Fotschki et al. (2018) [94] have reported the involvement of AhR in the hepatic immune-metabolic axis, including the upregulation of ANGPTL4. Together, these findings are indicative of a complex interplay among receptor pathways, suggesting that the 3D environment may enhance receptor crosstalk, thereby amplifying the cellular response to lower concentrations of toxicant. This could explain the increased sensitivity and earlier detection of genes like *Cyp1a1*, *Angptl4*, *Cd36*, and *Pgc1α*, which are regulated by multiple receptors and can be activated by many constituents found in a complex mixture such as NAFCs. Furthermore, there was an increase in conventional dose–response relationships observed in 3D cultures compared to 2D cultures. The more pronounced dose-dependent changes in gene expression in 3D cultures suggest a more similar response to *in vivo* responses and could be attributed to the increased complexity of cellular architecture, which may provide a more accurate representation of how cells in an organism would react to varying concentrations of a toxicant. Consequently, these results show that 3D cultures may be a more relevant model for studying the effects of environmental agents, especially at lower, environmentally relevant concentrations.

A key component of the cellular complexity in 3D models is the extracellular matrix (ECM). In cell cultures, the ECM is a complex mixture surrounding live cells, including the chemical compounds in the attachment surface of cell culture dishes, the media, and other structural and adhesive macromolecules that are essential in supplying stability and support to the cells [95]. Cells interact with the ECM through their respective receptors and exhibit crosstalk between receptor types. These interactions are primarily influenced by the physical properties and composition of the attachment surface, which can significantly impact the transcriptome and cell behavior [96,97]. However, in adherent 2D cultures, the role of ECM is limited to interactions between cells and the cell culture dish. Our study compares 3D and 2D mRNA expression across multiple receptor pathways, suggesting that the spatial arrangement in 3D cultures better facilitates attachment to the surface of cell culture dishes, which can influence the transcriptome. Recent literature suggests that in 3D drug-induced cell proliferation models, spheroids respond to complex cellular communication and behavior. We also analyzed mRNA expression of ECM regulatory elements, such as cadherins and connexins, and observed no significant changes following NAFC exposure. This finding suggests that ECM–cell interactions involve more than just transcriptional regulation of ECM components. The ECM’s composition, stiffness, and topography can influence cellular responses, contributing to the heightened sensitivity observed in the mRNA expression of NAFC-exposed 3D cultures at lower concentrations [25 mg/L]. As the ECM plays a crucial role in cellular interaction and response to apoptosis-inducing stimuli [34,98], several studies on cancer lines have reported an increase in apoptotic markers (Caspase 3, Caspase 7, and Caspase 9) in 3D cells compared to 2D cells. In fact, Kim et al. (2019) [99] observed a 3.7-fold increase in apoptosis in 3D cultures treated with XAV939, a response not evident in 2D cultures. Similarly, Keeratichamroen et al. (2023) [100] reported enhanced apoptosis in A549 cells under 3D culture conditions, as opposed to their 2D counterparts. However, it is important to note that while these studies demonstrate an increased sensitivity to apoptosis in the 3D model, both utilized matrigel/agar-coated plates for culturing their 3D spheroids and did not assess for necrotic core. One approach to mitigate this issue is the use of ULA-plates and scaffolds, which restrict the size of the spheroids, potentially reducing the formation of necrotic cores. In our study, NAFC exposure [25 and 125 mg/L] in 2D cultured McA-RH7777 cells induced caspase 3/7 activity, while NAFC exposure in 3D spheroids grown in ULA-plates showed no change, indicative of increased resistance of 3D spheroids to NAFC exposure compared to their 2D counterparts. Comparably, Imamura et al. (2015) [101] employed a 3D culturing technique similar to ours and found that cells in 2D cultures showed greater increases in cleaved-PARP expression following drug treatment compared to those in 3D culture. Together, these studies indicate that the structured 3D environment may offer protection against toxicant-induced apoptosis, with this protective effect potentially influenced by the specific methods used to culture 3D spheroids.

## 5. Conclusions

In summary, cultured hepatocytes are useful models for investigating the toxicity of drugs, including metabolism and biological and chemical mechanisms. Since xenobiotic receptors play a critical role in the metabolism and detoxification of drugs and environmental toxins, our study emphasizes the potential of 3D cultures as a platform for more accurately assessing the intricate interplay and communication between various signaling pathways, including receptor crosstalk and activation. In environmental risk assessment, the increased gene expression seen in 3D cultures may lead to better insight into chemical behavior and the *in vitro* response. As a generalized mammalian model, this platform could lead to more accurate assessments of potential health risks for wildlife, particularly for analyzing chemicals and complex environmental mixtures. Importantly, the observed increased gene expression in 3D cultures at the lower end of the exposure spectrum provides a better ability to discern the effects of compounds across a broader range of environmentally relevant doses and conditions without requiring high levels of exposure to observe significant effects. Thus, 3D hepatocyte cultures play a pivotal role in advancing our understanding of environmental toxicity.

## Figures and Tables

**Table 1:** Table of Primer Sequences.

Accession Number	Gene Name	Symbol	Forward Sequence (5′-3′)	Reverse Sequence (5′-3′)
NM_017101.1	Peptidylprolyl isomerase A	*Ppia*	CCGCTGTCTCTTTTCGCC	GCTGTCTTTGGAACTTTGTCTGC
NM_012512.2	beta-2-microglobulin	*B2m*	AATTCACACCCACCGAGACC	GCTCCTTCAGAGTGACGTGT
NM_012540.3	Cytochrome P450 Family 1 Subfamily A Member 1	*Cyp1a1*	TTGGGGAGGTTACTGGTTCTG	GAGTTAGGGAGGTAACGGAGG
XM_039102285.1	TCDD Inducible Poly(ADP-Ribose) Polymerase	*Tiparp*	GGTGGGATTGGGTTAAGCTCT	CAGTCCATCTATACACCTGCCC
XM_039079418.1	Angiopoietin Like 4	*Angptl4*	CAGGACTGGGATGGCAATG	CCTCACCCCCCAAATGG
XM_006230695.3	Carnitine Palmitoyltransferase 1A	*Cpt1a*	CACCCCAACCCATATCCAGG	TCCTCACGGTCTAATGTGCG
NM_031561.2	CD36 Molecule	*Cd36*	TCTCACACAACTCAGATACTGCT	GCACTTGCTTCTTGCCAACT
NM_001193568.1	Serum/Glucocorticoid Regulated Kinase 1	*Sgk1*	GAAACGGTCTTGCAGTGACG	CTGACTGACAACTGGGGCAT
NM_031345.1	TSC22 Domain Family Member 3	*Gilz*	GGAGGTCCTAAAGGAGCAGATTC	GCGTCTTCAGGAGGGTATTCTC
NM_031347.1	PPARG Coactivator 1 Alpha	*Pgc1* *α*	ATGGAGTGACATAGAGTGTGCT	CACCACTTCAATCCACCCAGA
NM_001173450.1	Fatty Acid Binding Protein 4	*Fabp4*	GCCCAAGCTGGGATCTCA	CCCCCATGAAAAGTCTTCTAGTAAAA
NM_012598.2	Lipoprotein Lipase	*Lpl*	AACCCCAGCAAGGCATACAG	AGGCAGAGCCCTTTCTCAAAT
NM_001004099.2	Gap Junction Protein Beta 2	*Cx26*	GCGACCCACTTCTGACCAA	TCCCCTGTAAGGAGCAGGT
NM_012567.2	Gap Junction Protein Alpha 1	*Cx43*	GCACTTTTCTTTCATTGGGGGA	GTCTGCTGTTTGGTACT

## Data Availability

The data presented in this study are available on request from the corresponding author.

## References

[1] Pampaloni F., Stelzer E.H. (2009). Three-Dimensional Cell Cultures in Toxicology. Biotechnol. Genet. Eng. Rev..

[2] Jensen C., Teng Y. (2020). Is It Time to Start Transitioning from 2D to 3D Cell Culture?. Front. Mol. Biosci..

[3] de Boo J., Hendriksen C. (2005). Reduction Strategies in Animal Research: A Review of Scientific Approaches at the Intra-experimental, Supra-experimental and Extra-experimental Levels. Altern. Lab. Anim..

[4] Franco N., Olsson I. (2014). Scientists and the 3Rs: Attitudes to animal use in biomedical research and the effect of mandatory training in laboratory animal science. Lab. Anim..

[5] Government of Canada Bill S-5, Strengthening Environmental Protection for a Healthier Canada Act—Summary of Amendments. https://www.canada.ca/en/services/environment/pollution-waste-management/strengthening-canadian-environmental-protection-act-1999/bill-c-28-strengthening-environmental-protection-healthier-canada-act-summary-amendments.html.

[6] Lelièvre S.A., Kwok T., Chittiboyina S. (2017). Architecture in 3D cell culture: An essential feature for in vitro toxicology. Toxicol. In Vitro.

[7] Stock K., Estrada M.F., Vidic S., Gjerde K., Rudisch A., Santo V.E., Barbier M., Blom S., Arundkar S.C., Selvam I. (2016). Capturing tumor complexity in vitro: Comparative analysis of 2D and 3D tumor models for drug discovery. Sci. Rep..

[8] Fang Y., Eglen R.M. (2017). Three-Dimensional Cell Cultures in Drug Discovery and Development. SLAS Discov. Adv. Sci. Drug Discov..

[9] Soldatow V.Y., LeCluyse E.L., Griffith L.G., Rusyn I. (2013). In vitro models for liver toxicity testing. Toxicol. Res..

[10] Sutherland R.M., Inch W.R., McCredie J.A., Kruuv J. (1970). A Multi-component Radiation Survival Curve Using an in Vitro Tumour Model. Int. J. Radiat. Biol. Relat. Stud. Phys. Chem. Med..

[11] Sutherland R.M., McCredie J.A., Inch W.R. (1971). Growth of multicell spheroids in tissue culture as a model of nodular carcinomas. J. Natl. Cancer Inst..

[12] Hamilton G.A., Westmorel C., George A.E. (2001). Effects of medium composition on the morphology and function of rat hepatocytes cultured as spheroids and monolayers. In Vitro Cell. Dev. Biol. Anim..

[13] Sharin T., Crump D., O’Brien J.M. (2020). Evaluation of the Aryl Hydrocarbon Receptor Response in LMH 3D Spheroids. Environ. Toxicol. Chem..

[14] Meng Q. (2010). Three-dimensional culture of hepatocytes for prediction of drug-induced hepatotoxicity. Expert Opin. Drug Metab. Toxicol..

[15] Guillouzo A. (1998). Liver cell models in in vitro toxicology. Environ. Health Perspect..

[16] Hu W.S., Friend J.R., Wu F.J., Sielaff T., Peshwa M.V., Lazar A., Nyberg S.L., Remmel R.P., Cerra F.B. (1997). Development of a bioartificial liver employing xenogeneic hepatocytes. Cytotechnology.

[17] Maurice M., Rogier E., Cassio D., Feldmann G. (1988). Formation of plasma membrane domains in rat hepatocytes and hepatoma cell lines in culture. J. Cell Sci..

[18] Jigorel E., Le Vee M., Boursier-Neyret C., Bertrand M., Fardel O. (2005). Functional expression of sinusoidal drug transporters in primary human and rat hepatocytes. Drug Metab. Dispos..

[19] Knop E., Bader A., Böker K., Pichlmayr R., Sewing K.-F. (1995). Ultrastructural and functional differentiation of hepatocytes under long-term culture conditions: Differentiation of Hepatocytes. Anat. Rec..

[20] Edmondson R., Broglie J.J., Adcock A.F., Yang L. (2014). Three-Dimensional Cell Culture Systems and Their Applications in Drug Discovery and Cell-Based Biosensors. ASSAY Drug Dev. Technol..

[21] Gunness P., Mueller D., Shevchenko V., Heinzle E., Ingelman-Sundberg M., Noor F. (2013). 3D Organotypic Cultures of Human HepaRG Cells: A Tool for In Vitro Toxicity Studies. Toxicol. Sci..

[22] Ramaiahgari S.C., den Braver M.W., Herpers B., Terpstra V., Commandeur J.N.M., van de Water B., Price L.S. (2014). A 3D in vitro model of differentiated HepG2 cell spheroids with improved liver-like properties for repeated dose high-throughput toxicity studies. Arch. Toxicol..

[23] Skardal A., Devarasetty M., Soker S., Hall A.R. (2015). In situ patterned micro 3D liver constructs for parallel toxicology testing in a fluidic device. Biofabrication.

[24] Bierwolf J., Lutgehetmann M., Feng K., Erbes J., Deichmann S., Toronyi E., Stieglitz C., Nashan B., Ma P.X., Pollok J.M. (2011). Primary rat hepatocyte culture on 3D nanofibrous polymer scaffolds for toxicology and pharmaceutical research. Biotechnol. Bioeng..

[25] Nguyen D.G., Funk J., Robbins J.B., Crogan-Grundy C., Presnell S.C., Singer T., Roth A.B. (2016). Bioprinted 3D Primary Liver Tissues Allow Assessment of Organ-Level Response to Clinical Drug Induced Toxicity In Vitro. PLoS ONE.

[26] Ingelman-Sundberg M., Lauschke V.M. (2022). 3D human liver spheroids for translational pharmacology and toxicology. Basic Clin. Pharmacol. Toxicol..

[27] Kammerer S. (2021). Three-Dimensional Liver Culture Systems to Maintain Primary Hepatic Properties for Toxicological Analysis In Vitro. Int. J. Mol. Sci..

[28] Lauschke V.M., Shafagh R.Z., Hendriks D.F.G., Ingelman-Sundberg M. (2019). 3D Primary Hepatocyte Culture Systems for Analyses of Liver Diseases, Drug Metabolism, and Toxicity: Emerging Culture Paradigms and Applications. Biotechnol. J..

[29] Serras A.S., Rodrigues J.S., Cipriano M., Rodrigues A.V., Oliveira N.G., Miranda J.P. (2021). A Critical Perspective on 3D Liver Models for Drug Metabolism and Toxicology Studies. Front. Cell Dev. Biol..

[30] Yang S., Ooka M., Margolis R.J., Xia M. (2023). Liver three-dimensional cellular models for high-throughput chemical testing. Cell Rep. Methods.

[31] Messner S., Agarkova I., Moritz W., Kelm J.M. (2013). Multi-cell type human liver microtissues for hepatotoxicity testing. Arch. Toxicol..

[32] Wrzesinski K., Magnone M.C., Hansen L.V., Kruse M.E., Bergauer T., Bobadilla M., Gubler M., Mizrahi J., Zhang K., Andreasen C.M. (2013). HepG2/C3A 3D spheroids exhibit stable physiological functionality for at least 24 days after recovering from trypsinisation. Toxicol. Res..

[33] Lee B.H., Kim M.H., Lee J.H., Seliktar D., Cho N.-J., Tan L.P. (2015). Modulation of Huh7.5 spheroid formation and functionality using modified PEG-based hydrogels of different stiffness. PLoS ONE.

[34] Takahashi Y., Hori Y., Yamamoto T., Urashima T., Ohara Y., Tanaka H. (2015). 3D spheroid cultures improve the metabolic gene expression profiles of HepaRG cells. Biosci. Rep..

[35] Fey S.J., Wrzesinski K. (2012). Determination of drug toxicity using 3D spheroids constructed from an immortal human hepatocyte cell line. Toxicol. Sci. Off. J. Soc. Toxicol..

[36] Sakai Y., Yamagami S., Nakazawa K. (2009). Comparative Analysis of Gene Expression in Rat Liver Tissue and Monolayer- and Spheroid-Cultured Hepatocytes. Cells Tissues Organs.

[37] Abu-Absi S.F., Friend J.R., Hansen L.K., Hu W.-S. (2002). Structural Polarity and Functional Bile Canaliculi in Rat Hepatocyte Spheroids. Exp. Cell Res..

[38] Brophy C.M., Luebke-Wheeler J.L., Amiot B.P., Khan H., Remmel R.P., Rinaldo P., Nyberg S.L. (2009). Rat hepatocyte spheroids formed by rocked technique maintain differentiated hepatocyte gene expression and function. Hepatology.

[39] Landry J., Bernier D., Ouellet C., Goyette R., Marceau N. (1985). Spheroidal aggregate culture of rat liver cells: Histotypic reorganization, biomatrix deposition, and maintenance of functional activities. J. Cell Biol..

[40] Kratschmar D.V., Messner S., Moritz W., Odermatt A. (2013). Characterization of a Rat Multi-Cell Type 3D-Liver Microtissue System. J. Tissue Sci. Eng..

[41] Frank R.A., Kavanagh R., Burnison B.K., Headley J.V., Peru K.M., Der Kraak G.V., Solomon K.R. (2006). Diethylaminoethyl-cellulose clean-up of a large volume naphthenic acid extract. Chemosphere.

[42] Marentette J.R., Frank R.A., Bartlett A.J., Gillis P.L., Hewitt L.M., Peru K.M., Headley J.V., Brunswick P., Shang D., Parrott J.L. (2015). Toxicity of naphthenic acid fraction components extracted from fresh and aged oil sands process-affected waters, and commercial naphthenic acid mixtures, to fathead minnow (*Pimephales promelas*) embryos. Aquat. Toxicol..

[43] Bartlett A.J., Frank R.A., Gillis P.L., Parrott J.L., Marentette J.R., Brown L.R., Hooey T., Vanderveen R., McInnis R., Brunswick P. (2017). Toxicity of naphthenic acids to invertebrates: Extracts from oil sands process-affected water versus commercial mixtures. Environ. Pollut..

[44] Björnsson E.S., Vucic V., Stirnimann G., Robles-Díaz M. (2022). Role of Corticosteroids in Drug-Induced Liver Injury. A Systematic Review. Front. Pharmacol..

[45] Flynn B.P., Birnie M.T., Kershaw Y.M., Pauza A.G., Kim S., Baek S., Rogers M.F., Paterson A.R., Stavreva D.A., Murphy D. (2021). Corticosterone pattern-dependent glucocorticoid receptor binding and transcriptional regulation within the liver. PLoS Genet..

[46] Wang S.-H., Liang C.-T., Liu Y.-W., Huang M.-C., Huang S.-C., Hong W.-F., Su J.-G.J. (2009). Crosstalk between activated forms of the aryl hydrocarbon receptor and glucocorticoid receptor. Toxicology.

[47] Rando G., Tan C.K., Khaled N., Montagner A., Leuenberger N., Bertrand-Michel J., Paramalingam E., Guillou H., Wahli W. (2016). Glucocorticoid receptor-PPARα axis in fetal mouse liver prepares neonates for milk lipid catabolism. eLife.

[48] Hasan A.U., Ohmori K., Hashimoto T., Kamitori K., Yamaguchi F., Rahman A., Tokuda M., Kobori H. (2018). PPARγ activation mitigates glucocorticoid receptor-induced excessive lipolysis in adipocytes via homeostatic crosstalk. J. Cell. Biochem..

[49] Yamamoto A., Kakuta H., Sugimoto Y. (2014). Involvement of glucocorticoid receptor activation on anti-inflammatory effect induced by peroxisome proliferator-activated receptor γ agonist in mice. Int. Immunopharmacol..

[50] Marentette J.R., Frank R.A., Hewitt L.M., Gillis P.L., Bartlett A.J., Brunswick P., Shang D., Parrott J.L. (2015). Sensitivity of walleye (*Sander vitreus*) and fathead minnow (*Pimephales promelas*) early-life stages to naphthenic acid fraction components extracted from fresh oil sands process-affected waters. Environ. Pollut..

[51] Clemente J.S., Prasad N.G.N., MacKinnon M.D., Fedorak P.M. (2003). A statistical comparison of naphthenic acids characterized by gas chromatography–mass spectrometry. Chemosphere.

[52] Quagraine E.K., Peterson H.G., Headley J.V. (2005). In Situ Bioremediation of Naphthenic Acids Contaminated Tailing Pond Waters in the Athabasca Oil Sands Region—Demonstrated Field Studies and Plausible Options: A Review. J. Environ. Sci. Health Part A.

[53] Vander Meulen I.J., Schock D.M., Parrott J.L., Mundy L.J., Pauli B.D., Peru K.M., McMartin D.W., Headley J.V. (2021). Characterization of naphthenic acid fraction compounds in water from Athabasca oil sands wetlands by Orbitrap high-resolution mass spectrometry. Sci. Total Environ..

[54] Jamshed L., Perono G.A., Yacoub L.R., Gutgesell R.M., Frank R.A., Hewitt L.M., Thomas P.J., Holloway A.C. (2022). The effects of oil sands process-affected water naphthenic acid fraction components on GDF15 secretion in extravillous trophoblast cells. Toxicol. Appl. Pharmacol..

[55] Monostory K., Kőhalmy K., Prough R.A., Kóbori L., Vereczkey L. (2005). The effect of synthetic glucocorticoid, dexamethasone on CYP1A1 inducibility in adult rat and human hepatocytes. FEBS Lett..

[56] Gomez A., Bindesbøll C., Satheesh S.V., Grimaldi G., Hutin D., MacPherson L., Ahmed S., Tamblyn L., Cho T., Nebb H.I. (2018). Characterization of TCDD-inducible poly-ADP-ribose polymerase (TIPARP/ARTD14) catalytic activity. Biochem. J..

[57] Xu A., Lam M.C., Chan K.W., Wang Y., Zhang J., Hoo R.L.C., Xu J.Y., Chen B., Chow W.-S., Tso A.W.K. (2005). Angiopoietin-like protein 4 decreases blood glucose and improves glucose tolerance but induces hyperlipidemia and hepatic steatosis in mice. Proc. Natl. Acad. Sci. USA.

[58] Staiger H., Haas C., Machann J., Werner R., Weisser M., Schick F., Machicao F., Stefan N., Fritsche A., Häring H.-U. (2009). Muscle-Derived Angiopoietin-Like Protein 4 Is Induced by Fatty Acids via Peroxisome Proliferator–Activated Receptor (PPAR)-δ and Is of Metabolic Relevance in Humans. Diabetes.

[59] Singh A.K., Chaube B., Zhang X., Sun J., Citrin K.M., Canfrán-Duque A., Aryal B., Rotllan N., Varela L., Lee R.G. (2021). Hepatocyte-specific suppression of ANGPTL4 improves obesity-associated diabetes and mitigates atherosclerosis in mice. J. Clin. Investig..

[60] Lichtenstein L., Berbée J.F.P., van Dijk S.J., van Dijk K.W., Bensadoun A., Kema I.P., Voshol P.J., Müller M., Rensen P.C.N., Kersten S. (2007). Angptl4 Upregulates Cholesterol Synthesis in Liver via Inhibition of LPL- and HL-Dependent Hepatic Cholesterol Uptake. Arterioscler. Thromb. Vasc. Biol..

[61] Yu C.-Y., Mayba O., Lee J.V., Tran J., Harris C., Speed T.P., Wang J.-C. (2010). Genome-Wide Analysis of Glucocorticoid Receptor Binding Regions in Adipocytes Reveal Gene Network Involved in Triglyceride Homeostasis. PLoS ONE.

[62] Liu X., Wang Y., Ortlund E.A. (2019). First High-Resolution Crystal Structures of the Glucocorticoid Receptor Ligand-Binding Domain–Peroxisome Proliferator-Activated γ Coactivator 1-α Complex with Endogenous and Synthetic Glucocorticoids. Mol. Pharmacol..

[63] Furuhashi M., Saitoh S., Shimamoto K., Miura T. (2014). Fatty Acid-Binding Protein 4 (FABP4): Pathophysiological Insights and Potent Clinical Biomarker of Metabolic and Cardiovascular Diseases. Clin. Med. Insights Cardiol..

[64] Sang Y., Kong P., Zhang S., Zhang L., Cao Y., Duan X., Sun T., Tao Z., Liu W. (2021). SGK1 in Human Cancer: Emerging Roles and Mechanisms. Front. Oncol..

[65] Ronchetti S., Migliorati G., Riccardi C. (2015). GILZ as a Mediator of the Anti-Inflammatory Effects of Glucocorticoids. Front. Endocrinol..

[66] Nataraja C., Dankers W., Flynn J., Lee J.P.W., Zhu W., Vincent F.B., Gearing L.J., Ooi J., Pervin M., Cristofaro M.A. (2021). GILZ Regulates the Expression of Pro-Inflammatory Cytokines and Protects Against End-Organ Damage in a Model of Lupus. Front. Immunol..

[67] Cannarile L., Delfino D.V., Adorisio S., Riccardi C., Ayroldi E. (2019). Implicating the Role of GILZ in Glucocorticoid Modulation of T-Cell Activation. Front. Immunol..

[68] Mittelbrunn M., Sánchez-Madrid F. (2012). Intercellular communication: Diverse structures for exchange of genetic information. Nat. Rev. Mol. Cell Biol..

[69] Bruzzone R., White T.W., Paul D.L. (1996). Connections with Connexins: The Molecular Basis of Direct Intercellular Signaling. Eur. J. Biochem..

[70] Stout R.F., Snapp E.L., Spray D.C. (2015). Connexin Type and Fluorescent Protein Fusion Tag Determine Structural Stability of Gap Junction Plaques. J. Biol. Chem..

[71] Crespo Yanguas S., Willebrords J., Maes M., da Silva T.C., Veloso Alves Pereira I., Cogliati B., Zaidan Dagli M.L., Vinken M. (2016). Connexins and pannexins in liver damage. EXCLI J..

[72] Li Y., Yang L., Tao R., Shang Y., Sun M., Peng S., Zhao G., Zhao Y. (2022). The Expression of Connexin 26 Regulates the Radiosensitivity of Hepatocellular Carcinoma Cells through a Mitogen-Activated Protein Kinases Signal Pathway. Int. J. Mol. Sci..

[73] Cogliati B., Da Silva T.C., Aloia T.P.A., Chaible L.M., Real-Lima M.A., Sanches D.S., Matsuzaki P., Hernandez-Blazquez F.J., Dagli M.L.Z. (2011). Morphological and molecular pathology of CCL4-induced hepatic fibrosis in connexin43-deficient mice. Microsc. Res. Tech..

[74] Livak K.J., Schmittgen T.D. (2001). Analysis of relative gene expression data using real-time quantitative PCR and the 2(-Delta Delta C(T)) Method. Methods.

[75] Quax R.A., Manenschijn L., Koper J.W., Hazes J.M., Lamberts S.W.J., van Rossum E.F.C., Feelders R.A. (2013). Glucocorticoid sensitivity in health and disease. Nat. Rev. Endocrinol..

[76] Hazra A., Pyszczynski N.A., DuBois D.C., Almon R.R., Jusko W.J. (2008). Modeling of corticosteroid effects on hepatic low-density lipoprotein receptors and plasma lipid dynamics in rats. Pharm. Res..

[77] Scheving L.A., Buchanan R., Krause M.A., Zhang X., Stevenson M.C., Russell W.E. (2007). Dexamethasone modulates ErbB tyrosine kinase expression and signaling through multiple and redundant mechanisms in cultured rat hepatocytes. Am. J. Physiol.-Gastrointest. Liver Physiol..

[78] Ayyar V.S., Almon R.R., DuBois D.C., Sukumaran S., Qu J., Jusko W.J. (2017). Functional proteomic analysis of corticosteroid pharmacodynamics in rat liver: Relationship to hepatic stress, signaling, energy regulation, and drug metabolism. J. Proteom..

[79] Steger D.J., Grant G.R., Schupp M., Tomaru T., Lefterova M.I., Schug J., Manduchi E., Stoeckert C.J., Lazar M.A. (2010). Propagation of adipogenic signals through an epigenomic transition state. Genes Dev..

[80] Jiang M., Zhang L., Ma X., Hu W., Chen Y., Yu M., Wang Q., Li X., Yin Z., Zhu Y. (2013). Tamoxifen inhibits macrophage FABP4 expression through the combined effects of the GR and PPARγ pathways. Biochem. J..

[81] Brennan-Speranza T.C., Henneicke H., Gasparini S.J., Blankenstein K.I., Heinevetter U., Cogger V.C., Svistounov D., Zhang Y., Cooney G.J., Buttgereit F. (2012). Osteoblasts mediate the adverse effects of glucocorticoids on fuel metabolism. J. Clin. Investig..

[82] Rafacho A., Roma L.P., Taboga S.R., Boschero A.C., Bosqueiro J.R. (2007). Dexamethasone-induced insulin resistance is associated with increased connexin 36 mRNA and protein expression in pancreatic rat islets. Can. J. Physiol. Pharmacol..

[83] Liu X., Qin J., Chang M., Wang S., Li Y., Pei X., Wang Y. (2020). Enhanced differentiation of human pluripotent stem cells into pancreatic endocrine cells in 3D culture by inhibition of focal adhesion kinase. Stem Cell Res. Ther..

[84] Clemente J.S., Fedorak P.M. (2005). A review of the occurrence, analyses, toxicity, and biodegradation of naphthenic acids. Chemosphere.

[85] Li C., Fu L., Stafford J., Belosevic M., Gamal El-Din M. (2017). The toxicity of oil sands process-affected water (OSPW): A critical review. Sci. Total Environ..

[86] Gutgesell R.M., Jamshed L., Frank R.A., Hewitt L.M., Thomas P.J., Holloway A.C. (2022). Naphthenic acid fraction components from oil sands process-affected water from the Athabasca Oil Sands Region impair murine osteoblast differentiation and function. J. Appl. Toxicol..

[87] Kassotis C.D., Nagel S.C., Stapleton H.M. (2018). Unconventional oil and gas chemicals and wastewater-impacted water samples promote adipogenesis via PPARγ-dependent and independent mechanisms in 3T3-L1 cells. Sci. Total Environ..

[88] Lister A., Nero V., Farwell A., Dixon D.G., Van Der Kraak G. (2008). Reproductive and stress hormone levels in goldfish (Carassius auratus) exposed to oil sands process-affected water. Aquat. Toxicol. Amst. Neth..

[89] Sérée E., Villard P.-H., Pascussi J.-M., Pineau T., Maurel P., Nguyen Q.B., Fallone F., Martin P.-M., Champion S., Lacarelle B. (2004). Evidence for a new human CYP1A1 regulation pathway involving PPAR-α and 2 PPRE sites. Gastroenterology.

[90] Tan M.J., Teo Z., Sng M.K., Zhu P., Tan N.S. (2012). Emerging Roles of Angiopoietin-like 4 in Human Cancer. Mol. Cancer Res..

[91] Oike Y., Akao M., Kubota Y., Suda T. (2005). Angiopoietin-like proteins: Potential new targets for metabolic syndrome therapy. Trends Mol. Med..

[92] Smati S., Régnier M., Fougeray T., Polizzi A., Fougerat A., Lasserre F., Lukowicz C., Tramunt B., Guillaume M., Burnol A.-F. (2020). Regulation of hepatokine gene expression in response to fasting and feeding: Influence of PPAR-α and insulin-dependent signalling in hepatocytes. Diabetes Metab..

[93] Xu J., Chen W., Wang Z., Xin M., Gao S., Liu W., Wang K., Ma J., Yan X., Ren Y. (2022). Profiles of transcriptome and metabolic pathways after hypobaric hypoxia exposure. Proteome Sci..

[94] Fotschki B., Laparra J.M., Sójka M. (2018). Raspberry Polyphenolic Extract Regulates Obesogenic Signals in Hepatocytes. Molecules.

[95] Assunção M., Dehghan-Baniani D., Yiu C.H.K., Später T., Beyer S., Blocki A. (2020). Cell-Derived Extracellular Matrix for Tissue Engineering and Regenerative Medicine. Front. Bioeng. Biotechnol..

[96] Salehi M.S., Neumann I.D., Jurek B., Pandamooz S. (2021). Co-Stimulation of Oxytocin and Arginine-Vasopressin Receptors Affect Hypothalamic Neurospheroid Size. Int. J. Mol. Sci..

[97] Totaro A., Panciera T., Piccolo S. (2018). YAP/TAZ upstream signals and downstream responses. Nat. Cell Biol..

[98] Yamada K.M., Pankov R., Cukierman E. (2003). Dimensions and dynamics in integrin function. Braz. J. Med. Biol. Res..

[99] Kim D., Koh B., Kim K.R., Kim K.Y., Jung W.H., Kim H.Y., Kim S., Rhee S.D. (2019). Anticancer effect of XAV939 is observed by inhibiting lactose dehydrogenase A in a 3-dimensional culture of colorectal cancer cells. Oncol. Lett..

[100] Keeratichamroen S., Sornprachum T., Ngiwsara L., Ornnork N., Svasti J. (2023). p-STAT3 influences doxorubicin and etoposide resistance of A549 cells grown in an *in vitro* 3D culture model. Oncol. Rep..

[101] Imamura Y., Mukohara T., Shimono Y., Funakoshi Y., Chayahara N., Toyoda M., Kiyota N., Takao S., Kono S., Nakatsura T. (2015). Comparison of 2D- and 3D-culture models as drug-testing platforms in breast cancer. Oncol. Rep..

